# Directed biomechanical compressive forces enhance fusion efficiency in model placental trophoblast cultures

**DOI:** 10.1038/s41598-024-61747-3

**Published:** 2024-05-17

**Authors:** Prabu Karthick Parameshwar, Chen Li, Kaline Arnauts, Junqing Jiang, Sabra Rostami, Benjamin E. Campbell, Hongyan Lu, Derek Hadar Rosenzweig, Cathy Vaillancourt, Christopher Moraes

**Affiliations:** 1 Department of Biological and Biomedical Engineering, McGill University, Montréal, Québec Canada; 2 Department of Chemical Engineering, McGill University, Montréal, Québec Canada; 3 Department of Surgery, McGill University, Montréal, Québec Canada; 4 Injury, Repair and Recovery Program, Research Institute of the McGill University Health Centre, Montréal, Québec Canada; 5 Institut National de la Recherche Scientifique (INRS)-Centre Armand-Frappier Santé Biotechnologie, Laval, Québec Canada; 6 Department of Obstetrics and Gynecology, Université de Montréal, and Research Center Centre Intégré Universitaire de Santé et de Services Sociaux (CIUSSS) du Nord-de-L’Île-de-Montréal, Montréal, Québec Canada; 7 Goodman Cancer Research Centre, McGill University, Montréal, Québec Canada; 8 Division of Experimental Medicine, McGill University, Montréal, Québec Canada

**Keywords:** Placenta, Choriocarcinoma, Fusion, Mechanics, Compression, Biophysical methods, Biomedical engineering

## Abstract

The syncytiotrophoblast is a multinucleated structure that arises from fusion of mononucleated cytotrophoblasts, to sheath the placental villi and regulate transport across the maternal–fetal interface. Here, we ask whether the dynamic mechanical forces that must arise during villous development might influence fusion, and explore this question using in vitro choriocarcinoma trophoblast models. We demonstrate that mechanical stress patterns arise around sites of localized fusion in cell monolayers, in patterns that match computational predictions of villous morphogenesis. We then externally apply these mechanical stress patterns to cell monolayers and demonstrate that equibiaxial compressive stresses (but not uniaxial or equibiaxial tensile stresses) enhance expression of the syndecan-1 and loss of E-cadherin as markers of fusion. These findings suggest that the mechanical stresses that contribute towards sculpting the placental villi may also impact fusion in the developing tissue. We then extend this concept towards 3D cultures and demonstrate that fusion can be enhanced by applying low isometric compressive stresses to spheroid models, even in the absence of an inducing agent. These results indicate that mechanical stimulation is a potent activator of cellular fusion, suggesting novel avenues to improve experimental reproductive modelling, placental tissue engineering, and understanding disorders of pregnancy development.

## Introduction

The placenta is a dynamic organ that serves as the main interface between the mother and the developing fetus^1,2^. The chorionic placental villus is the fundamental unit of this interface and consists of an outer multinucleated syncytiotrophoblast layer, which arises from fusion of subjacent mononuclear cytotrophoblasts^3,4^. Trophoblast fusion is hence critical for a successful pregnancy as the syncytiotrophoblast regulates transport between mother and fetus, and dysregulated fusion is associated with abnormal placentation that can lead to significant obstetric complications such as pre-eclampsia and intrauterine growth restriction^5^. While several models exist with which to study placental transport^6,7^ including recent advanced ‘on-a-chip’ devices^8–13^, creating perfectly fused syncytial sheets remains challenging in culture. While novel pluripotent stem cell models do provide one avenue to enhance fusion efficiencies^14–16^, developing a better understanding of the fundamental factors influencing syncytialization could further improve upon these advanced models, and enhance our understanding of the factors driving disease progression during pregnancy.

The chorionic villus begins developing as a small bud and progresses towards a branched terminal villus encapsulated within a syncytialized trophoblast sheath, through a process of repeated budding morphogenesis^17,18^. Syncytin, cytokines, proteases, growth factors and oxygen levels play a well-established role in successful fusion^19–21^. Conversely, the biochemical factors encountered during diseases including intrauterine growth restriction, gestational diabetes, and pre-eclampsia are known to disrupt fusion and impair villous formation^22^. While the effects of these cues on fusion processes have been well-studied, the impact of biomechanical factors on fusion, particularly those that arise during villous development, remains less clear.

Mechanical forces arising from budding morphogenesis have been established to play an important role in directing cell function in the gut^23^ and lungs^24,25^ for example; and are known to affect fusion in systems such as muscle^26^ and bone^27^. Such stress patterns can spontaneously arise during 3D tissue morphogenesis^28–30^ to directly impact cell function^31^. In placental models specifically, endogenous mechanical stress patterns have been computationally predicted to arise from trophoblast proliferation and fusion^32^, and such endogenous stresses have been experimentally shown to affect trophoblast fusion via models that tune substrate stiffness and colony sizes^33–35^. Hence, it seems likely that the spatially dynamic biomechanical stress profiles that arise during villous development could play an important role in the fusion process.

In this work, we ask whether fusion is associated with altered biomechanical stresses and show that sites of fusion in a trophoblast model of monolayer fusion are accompanied by spatial patterns of mechanical stress consistent with those expected from the development of bud-like projections. We then recreate these patterns of dynamic stress and demonstrate that the application of externally generated, spatially directed compressive stresses, similar to those that have been computational predicted to occur during budding morphogenesis of the placental villous tree, enhances fusion efficiency. We extend these findings to a 3D spheroid culture model, and demonstrate that low levels of compressive stress can enhance fusion even without a biochemical induction method of forskolin^36^.

## Results

### Increased compressive radial stresses arise at sites of induced fusion in culture

**Figure 1:**
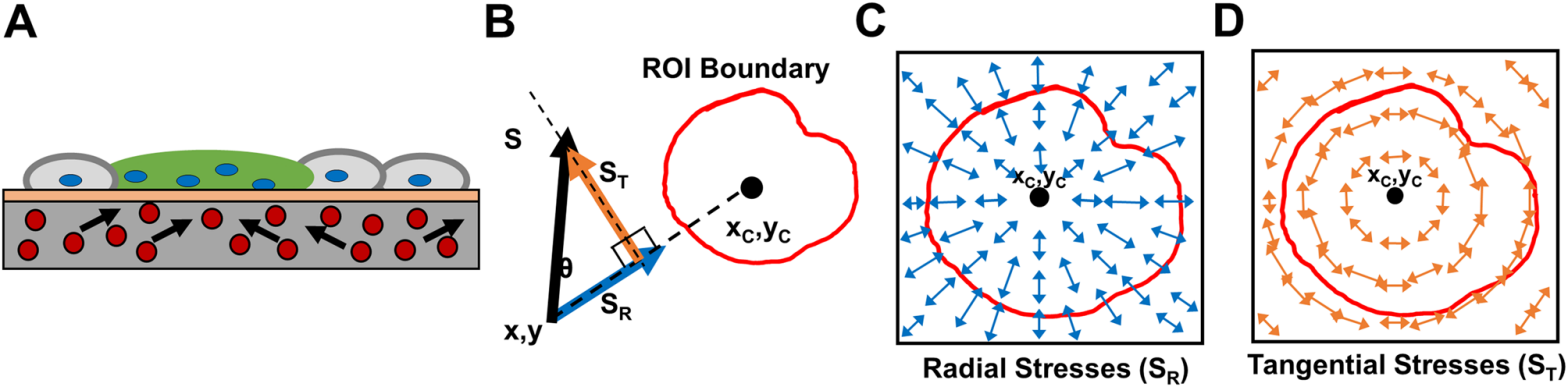
Techniques used for traction force microscopy (TFM) analysis. (**A**) Schematic illustrating the TFM experiment performed with sheets of fused and non-fused BeWo cells on a polyacrylamide gel. Arrows indicate the change in position of fluorescent beads that is used to calculate stresses. (**B**) Stress vectors *S* were decomposed into radial *S*_*R*_ (blue) and tangential *S*_*T*_ (orange) components based on the areal centroid (x_C_, y_C_) of a fused region. (**C**,**D**) Schematic illustrating an idealized stress field of all the correspondingly resolved radial and tangential stress vectors, respectively within and around a region of interest (ROI).

**Figure 2:**
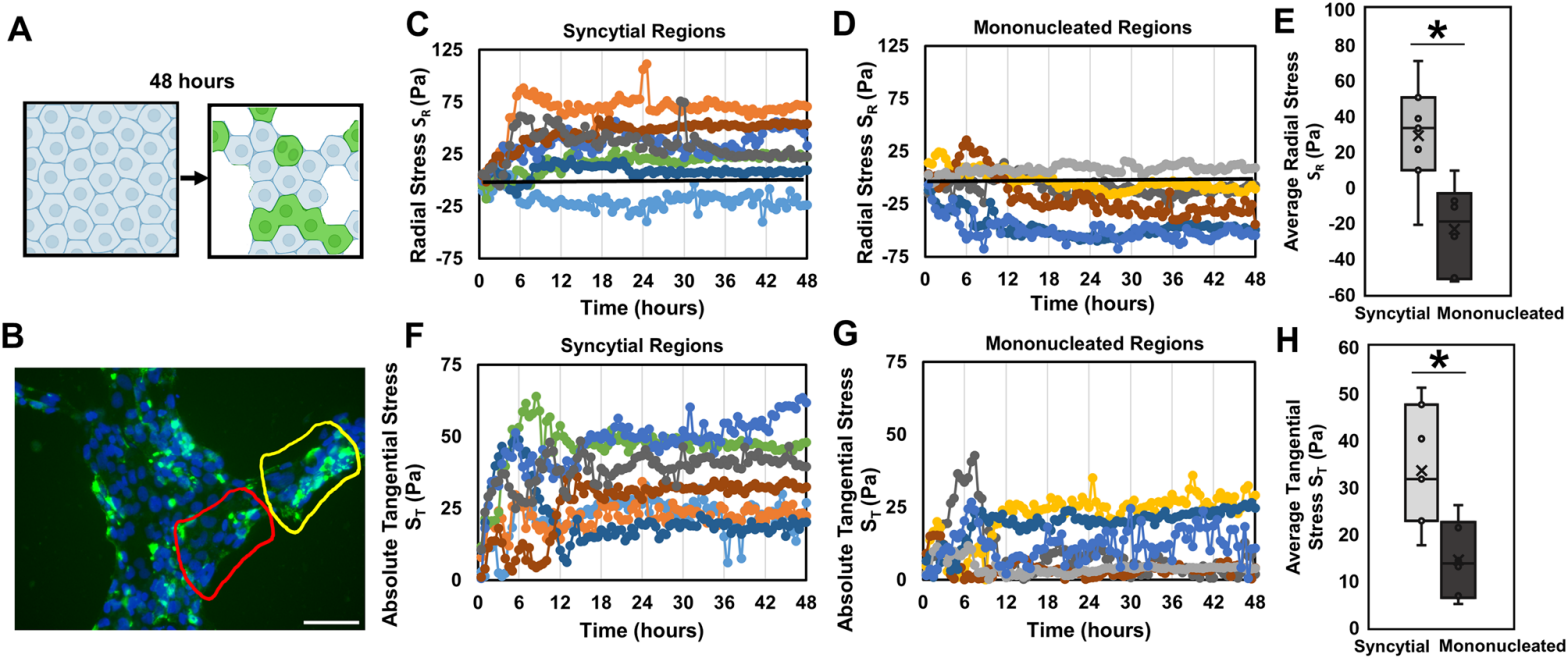
Traction force microscopy (TFM) performed on fusion induced BeWo monolayers. (**A**) Schematic depicting a sheet of cells undergoing fusion over a period of 48 h; fused regions represented in green. (**B**) Representative fluorescent image of a sheet of BeWo cells, showing a fused syncytial region (yellow outline) and a non-fused mononucleated region (red outline). Scale bar is 100 µm; green: Syndecan-1, blue: nuclei. (**C**,**D**) Radial traction stresses and (**F**,**G**) tangential traction stresses over 48 h, for both syncytial and mononucleated regions, respectively. Each line represents one tracked region. (**E**) Radial traction stresses and (**H**) tangential stresses, averaged from hours 12 to 48 for both syncytial and mononucleated regions. (Data presented as box plot distributions; the central line is the median, the box represents 25th to 75th percentile (lower and upper quartiles) and whiskers represent min/max values; n = 6–7 regions; *p < 0.05 by independent Student’s *t*-test).

While the mechanical impact of processes such as proliferation and apoptosis have previously been understood in biological systems, the mechanical features that accompany a trophoblast fusion event have not been previously studied. Here, using traction force microscopy (TFM), we aimed to quantitatively measure the stresses associated with stochastic fusion events observed after forskolin-induction in a classical choriocarcinoma cell model system of trophoblast fusion (Figs. 1A and 2A). A live monolayer of BeWo cells was imaged under forskolin stimulation, and assessed for fusion by identifying those regions positive for Syndecan-1, a marker for successful fusion^37,38^. In this way, regions exhibiting fusion can be distinguished from those that remain mononucleated, and the history of mechanical stresses in those regions can be quantified.

As expected, we observed stochastic appearance of small fused (syncytial) patches across the fenestrated monolayer, amidst non-fused (mononucleated) regions (Fig. 2B; overall fusion efficiency of 38 ± 3% across 3 independent samples). We then considered stresses encountered during the prior 48 h around syncytial and mononucleated areas, and quantified the radial and tangential components of these stresses in relation to the centroid of the syncytial or mononucleated patch (Fig. 1B–D; Supplementary Fig. S1). This approach allowed us to quantify compression and tension in the radial (inwards/outwards) direction (radial stresses or S_R_) as well as circumferential stresses around the edges (tangential stresses or S_T_).

Changes in both radial and tangential traction stresses were observed within ~ 6 to 12 h after induction of fusion and then stayed relatively constant for the duration of the experiment (Fig. 2C,D,F,G). To quantify the mechanical stress levels without capturing this transition period, stresses from 12 to 48 h were averaged for comparison purposes (Fig. 2E,H). A statistically significant increase in compressive radial stresses (directed into the region of interest) was observed around syncytialized regions, but not in mononucleated sites of equivalent area (Fig. 2E). Similarly, an increase in tangential stresses (directed around the region of interest) was observed (Fig. 2F–H) for syncytialized regions. This pattern of biaxial compression towards a radial region matches the stresses predicted to arise during villous bud formation due to local proliferation of cells during morphogenesis^17,34^, suggesting that the process of fusion itself may also contribute to sculpting the villous bud in situ. However, whether these forces that arise in situ are merely correlated with, or a necessary component of the fusion process remains unclear and can only be addressed by recreating these dynamic mechanical stresses in culture.

### Externally applied uniaxial compression does not affect fusion efficiency

**Figure 3:**
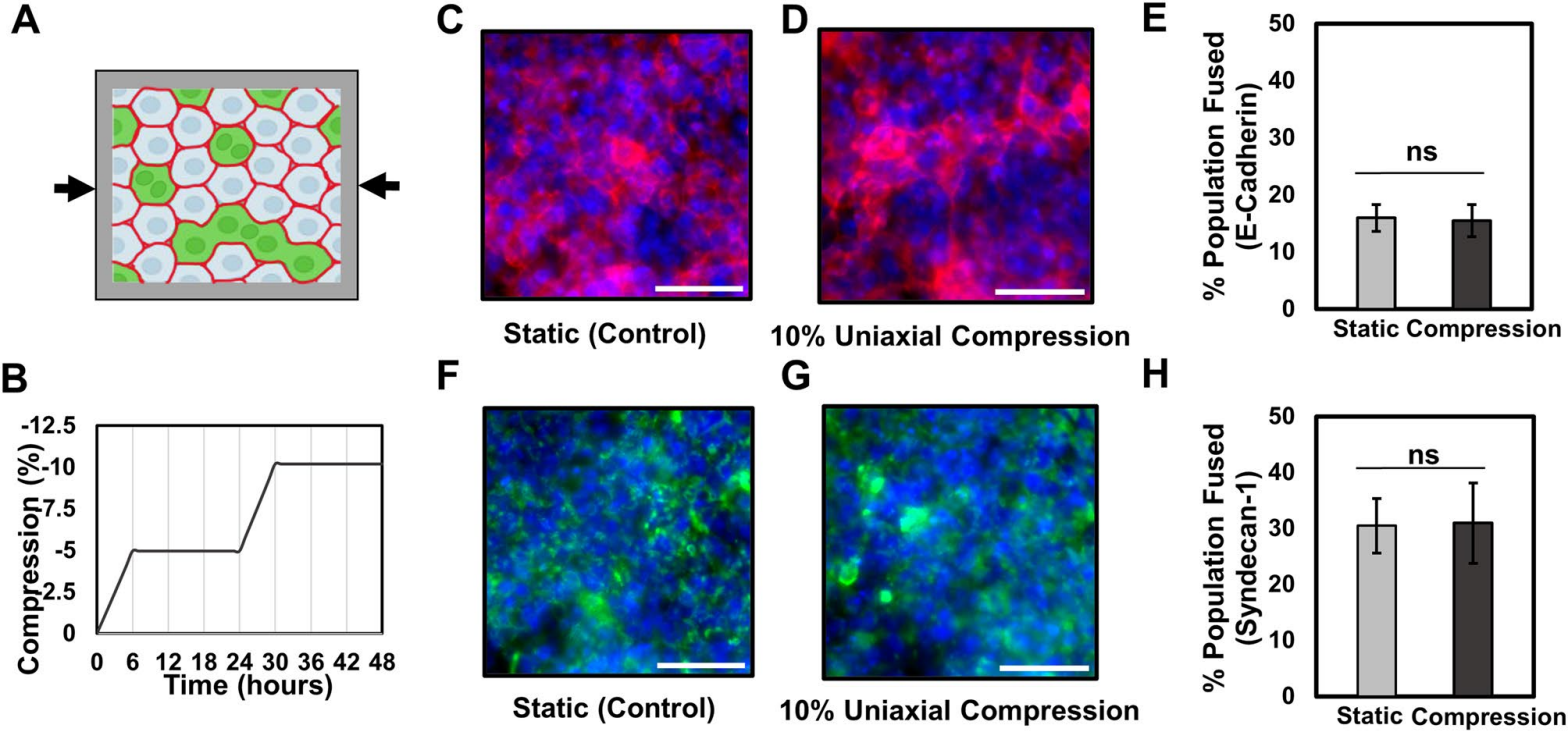
Effect of uniaxial compression on fusion in BeWo cells, induced with forskolin (24 µM) for 2 days. (**A**) Schematic illustrating the 10% uniaxial compression experiment performed along with a graph (**B**) showing the actual regimen over 48 h. (**C**,**D**) Fluorescent images of fused BeWo cells stained for E-cadherin junctional proteins, and (**F**,**G**) Syndecan-1 on static vs. 10% uniaxial compression over 2 days. Scale bar is 100 µm; green: Syndecan-1, red: E-cadherin, blue: nuclei. (**E**,**H**) Quantification of fusion efficiency calculated using E-cadherin and Syndecan-1, respectively. (Data presented as mean ± standard deviation; n = 5 independent samples, *ns*  not significant, p = 0.916 and p = 0.788, respectively by independent Student’s *t*-test).

To externally apply dynamic mechanical stresses to cells in monolayer culture, we utilized a commercially available elastomeric platform (CellScale MechanoCulture FX) to apply an externally defined strain-based deformation over time. The substrate strains are then expected to apply stresses to the adherent cells, which can then respond and remodel in response to the applied load. We first tested external uniaxial compressive strains, which should apply an appropriate load without matching the spatial patterns of stress observed in the stochastic fusion stress measurement experiments. A loading profile of 10% compression was selected based on the displacements observed in the traction force microscopy experiments; and applied in steps over two days (Fig. 3A,B). As negligible fusion was observed for BeWo cells in standard media, we added forskolin to all cultures for these experiments to compare the relative change in fusion efficiency due to compression. Interestingly, we found that uniaxial compression did not have any effect of fusion efficiency compared to a mechanically static control, based on analysis of the fraction of nuclei in fused syncytia, as assessed by the presence of an intact E-cadherin junctional network (Fig. 3C–E). We further confirmed that the fraction of nuclei in regions positive for Syndecan-1 expression did not change between mechanically static and uniaxial compression conditions (Fig. 3F–H). This would suggest that compression along a single axis is not sufficient to induce fusion. This is perhaps due to cell reorganization along an orthogonal axis to relieve the unidirectional stresses applied within the cell sheet. If true, this would further suggest that the spatial pattern of force is a key parameter in achieving high levels of fusion in this model.

### Equibiaxial compression increases fusion efficiency whereas equibiaxial tension does not

**Figure 4:**
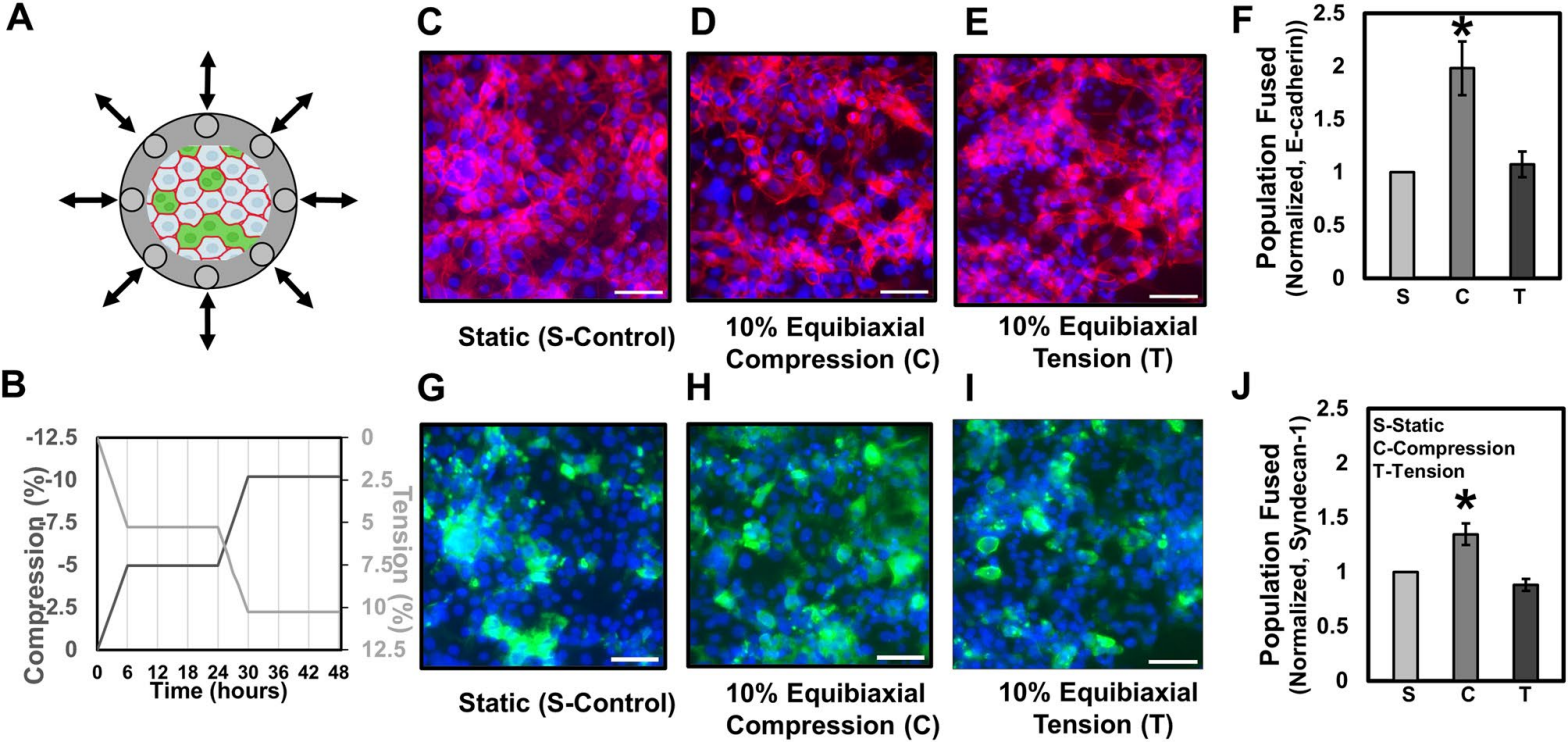
Effect of equibiaxial compression and tension on fusion in BeWo cells, induced with forskolin (24 µM) for 2 days. (**A**) Schematic illustrating the 10% equibiaxial compression/tension experiment performed along with a graph (**B**) showing the actual regimen over 48 h. (**C**–**E**) Fluorescent images of fused BeWo cells stained for E-cadherin junctional marker expression and (**G**-**I**) Syndecan-1 expression on static control, 10% uniaxial compression, and 10% equibiaxial tension substrates over 2 days. Scale bar is 100 µm; green: Syndecan-1, red: E-cadherin, blue: nuclei. (**F**,**J**) Quantification of averaged normalized population fused calculated using E-cadherin and Syndecan-1 expression. (Data presented as mean ± standard deviation; n = 3 independent experiments with 10 image viewfields each, sampled at random locations, *p < 0.05 by one-way ANOVA).

In order to apply mechanical compression and tension along both the radial and circumferential directions of a circular elastomeric cell culture substrate (Fig. 4A), we used a custom iris-like equibiaxial stretching system^39^. To control for possible confounding effects of substrate stiffness in these experiments, we fabricated a stiffness-matched custom silicone mixture in a petri dish to serve as a static control. No statistically significant differences in mechanical stiffness were observed between the commercially prefabricated stretch dishes and cast silicone elastomer static petri dish samples (Supplementary Fig. S2A–E), but this is likely due to the considerable variation observed across the samples tested. However, it is important to note that since substrate stiffnesses greater than 50 kPa have been shown to induce tissue culture plastic-like morphological phenotypes in BeWo cells^33^, these substrates can be considered sufficiently stiff for comparative purposes. Together, this data demonstrates that the control conditions can be considered as stiffness-equivalent substrates so as to isolate the effects of externally applied mechanical stress on the cell cultures. Furthermore, to control for any potential confounding effects of cellular density or space restriction in affecting fusion, we adjusted the initial seeding densities such that the final nuclear densities after mechanical deformation were similar (Supplementary Fig. S2F).

Similar to the uniaxial compression experiment, BeWo cells were subjected to 10% equibiaxial strains in either compression or tension over 2 days (Fig. 4B). A statistically significant increase in fusion was observed by analysis of both E-cadherin expression (Fig. 4C–F; Supplementary Table S1), and Syndecan-1 expression (Fig. 4G–J; Supplementary Table S2) only in equibiaxial compression conditions, and not in equibiaxial tension conditions. Taken together, these results confirm that for mechanical stimulation to enhance fusion, the stress fields must recreate both the directional (compressive) and spatial (biaxial) stresses as those predicted computationally^34^ and experimentally observed to arise spontaneously during stochastic fusion in monolayer culture (Fig. 2C,F).

### 3D compression confirms mechanical forces from compression positively influence fusion

To determine whether our findings extend to 3D culture models, we investigated the effect of compression on syncytiotrophoblast formation in a spheroidal BeWo model. Trophoblast organoids have recently emerged as important tools to study various aspects of placental biology including self-assembly, expansion and differentiation^15,16,35,40^. However in these models, fusion takes place at the core of the spheroids which is opposite to the in vivo situation^15,16^*.* We have previously shown that the spheroid core is subjected to compressive stresses generated by a ‘skin’ of tension formed on the spheroid surface^28^. Hence, we wondered whether the state of internal compression present in trophoblast organoids might drive internal syncytialization and sought to manipulate the internal compression in such systems.

**Figure 5:**
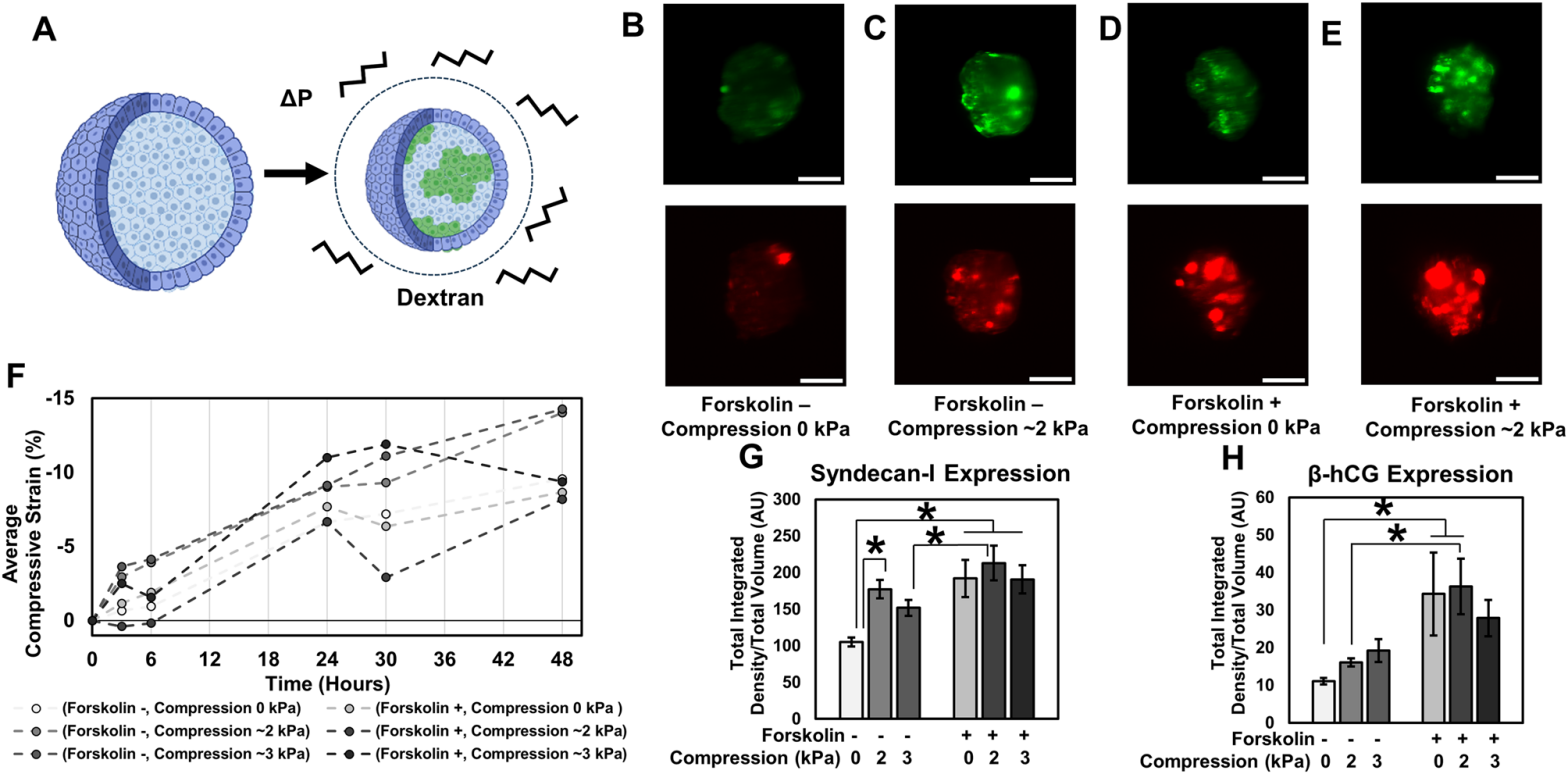
Effect of 3D compression on fusion in BeWo spheroids, induced with ( +)/without (-) forskolin (24 µM) and/or dextran for 2 days. (**A**) Schematic illustrating the 3D compression experiment performed using dextran molecules. (**B**–**E**) Representative fluorescent images of a slice of BeWo cells from spheroids subjected to various conditions. Scale bar is 100 µm; green: Syndecan-1, red: β-hCG. (**F**) Trend of quantified average compressive strain in spheroids subjected to various conditions at several time points over 48 h. (**G**,**H**) Quantification of fusion using Syndecan-1 and β-hCG markers. (Data presented as mean ± standard deviation; n = 3 spheroids per condition, *p < 0.05 by two-way ANOVA with post-hoc Tukey’s test).

To experimentally manipulate compressive stresses in 3D culture, we subjected BeWo spheroids to osmotically induced compression (Fig. 5A). Briefly, long-chain molecules in the cell culture media such as dextran are able to alter the osmotic pressure of a solution. Biological objects such as spheroids permit water transport but prevent dextran movement. This establishes an osmotic pressure gradient across the tissue boundary, through which the tissue undergoes compression in an isotropic stress field. This has been previously shown to significantly affect biological systems through processes such as reduced proliferation^41^ or Wnt-mediated stem cell renewal^42^. This technique has the advantage of generating mechanical stresses without complex equipment or structures that is often required for physical manipulation of 3D cultures.

To quantify the degree of compression generated by a known osmolar imbalance, we first determined the compressive strain exhibited by a polyacrylamide hydrogel of known stiffness (G = 5.15 ± 0.34 kPa, as assessed by shear rheometry) in response to 25 and 50 mg/mL of dextran. Finite element modelling was then used to determine the stresses required to achieve these deformations (1.85 ± 0.67 kPa or ~ 2 kPa, and 2.86 ± 2.50 kPa or ~ 3 kPa for 25 and 50 mg/mL, respectively). These values are consistent with other studies^43^.

BeWo spheroids were formed in polyacrylamide micropockets^44^ and maintained in culture for two days with and without both forskolin and externally applied compression (Fig. 5B–E). In all cases, spheroidal tissues exhibited some degree of additional compaction over two days, likely due to continued internal remodelling^28^. However, this compaction was markedly increased in spheroids subjected to dextran compression without forskolin treatment (Fig. 5F). Analysis of nuclear shape in immunostained sections of these tissues indicated that all nuclei were intact and normal (Supplementary Fig. S3), confirming that compression was achieved without inducing apoptosis or necrosis within the spheroidal tissue.

Analysis of fusion via E-cadherin junctional analysis was not possible in these models due to the densely packed nuclei, and so we evaluated fusion efficiency via whole-mount light sheet fluorescent analysis of Syndecan-1 expression (Fig. 5G–H), and further confirmed our findings with immunostained tissue sections (Supplemental Fig. S3). Although it remains a possibility that Syndecan-1 expression can occur without fusion, our comparative findings (Figs. 3, 4) suggest that this is unlikely. To obtain an unbiased estimate of the amount of Syndecan-1 expressed within the spheroids, the total integrated fluorescent intensity throughout the spheroid was assessed and normalized against spheroid volume. While the least fusion was observed in the condition without compression or forskolin, we noted that BeWo cells did exhibit some degree of fusion even without forskolin induction, in contrast with our studies of fusion in monolayers which required forskolin. We also noted that ~ 2 kPa of compression was sufficient to significantly increase fusion in standard cultures, whereas ~ 3 kPa stress did not have a similar effect, suggesting that low levels of compression optimally impact fusion. This seems reasonable as too high a compressive stress must adversely affect cell function and activity. Furthermore, at these levels of compression, we achieved fusion efficiencies statistically similar to those achieved via forskolin induction. Surprisingly, while intracellular levels of β-hCG did increase significantly with both compression and forskolin-induction, they did not match the levels of fusion inferred by Syndecan-I expression, consistent with other reports suggesting that intracellular β-hCG levels do not scale with fusion efficiency^33,45^. This data does however confirm that compression affects fusion in 3D, even in the absence of forskolin induction.

## Discussion

While mechanical forces are now well-established to play an important role in directing cell function^46^, the specific relationships between forces arising during development and the corresponding specification of cell behaviour are highly diverse across organ systems. Placental villous tree formation is a multifaceted and dynamic process, and understanding the precise mechanical contributions and impact of the various processes occurring during villous morphogenesis remains challenging. Here, we demonstrate that the process of fusion itself is associated with specific patterns of local stress at the site of fusion. These findings are consistent with other studies of cell fusion in muscles^47^ in which the highest contractile stresses were observed at the tips of cells undergoing differentiation. Interestingly, the patterns of stress observed in trophoblast fusion are similar to those that would drive morphogenetic budding in the placental bed. We then demonstrate that applying qualitatively similar patterns of external compressive stress can enhance cell fusion efficiency, in both 2D and 3D culture models. These results together suggest that a positive feedback loop exists between trophoblast fusion and mechanical remodelling that act together to sculpt a villous tree structure with a continuous syncytialized monolayer.

We also demonstrate that in 3D cultures, externally applied low levels of compressive stresses can potentially achieve similar levels of fusion as via chemical induction with forskolin. Although BeWo choriocarcinoma cells are a frequently used model for studies of trophoblast fusion, non-physiological chemical induction is often considered essential for fusion in this model. Speculatively, our findings therefore could suggest that downstream effects of chemical stimulation may be physically responsible for fusion. For example, forskolin induction increases production of cyclic adenosine monophosphate (cAMP) which is also known to regulate mechanical contractility in the heart^36,48^, and might establish pro-fusion mechanical conditions. This suggests the intriguing possibility that recently-developed trophoblast stem cell models^14,49^ that exhibit higher frequencies of spontaneous fusion may present distinct mechanical behaviours and architectures that make fusion more efficient.

Our study does have some significant technical considerations that limit interpretation of results. Specifically, analyzing fusion in 3D is limited to a volumetric estimate of total Syndecan-1 expression, which although frequently used in 3D models may not necessarily be definitive to assess fusion efficiency within a spheroid. Furthermore, quantitatively recreating the stress patterns measured via TFM and predicted via existing computational models cannot be achieved with current commercially available systems. 2D monolayer mechanical stimulation systems are strain-controlled, rather than stress-controlled, and rely on transfer of deformation patterns from the elastomeric culture substrate to the adherent cells. Conversely, osmotic compression of 3D systems is stress-controlled, but cannot create specific spatially defined deformation patterns. Hence, although our findings correlate well with each other and are conceptually unified, establishing quantitative stress responses could be an important next step for this work. This would require novel technological developments in cell mechanobiology stimulation platforms, perhaps using 3D engineered hydrogel model systems to apply local stresses to living tissue^50^.

More broadly, this specific study does have conceptual limitations that should be considered carefully. First, all experiments were performed with the BeWo choriocarcinoma cell line, which although a well-established model to study fusion processes specifically, may not accurately capture other aspects of placental biology. This is appropriate for a first study because we focus explicitly on establishing external mechanical stresses as fundamental drivers of fusion, but extending this work towards other trophoblast stem cell types would be an important next step. Second, the 2D experiments performed here examine fusion between adjacent cells in culture. Typically, maintenance of the syncytiotrophoblast requires fusion through the basal surface of an established syncytium, which is experimentally quite challenging to recreate. While we can conclude that in vitro fusion is impacted by mechanics, whether this translates directly to in vivo contexts such as syncytial maintenance or to primary syncytialization remains unclear. Finally, establishing causative mechanistic relationships between external mechanical stresses and fusion is particularly challenging. Unlike molecular systems, “external mechanics” cannot be specifically and precisely inhibited or targeted without affecting a wide variety of cellular processes. Nevertheless, establishing the mechanosensitive pathways that affect fusion would be an important next step in identifying actionable mechanisms to manipulate fusion as needed.

In summary, our results demonstrate that directed external mechanical stresses in the form of compression, such as those that would arise when a small patch of cells fuse, might be an important factor in efficient fusion in vitro. We also explicitly show this in spheroids where greater fusion was observed under low compressive stresses even in the absence of forskolin, demonstrating that mechanical stimulation is likely a potent stimulator of fusion and can be utilized as a tool to impact in vitro levels of fusion. Broadly, this work provides a better mechanobiological understanding of trophoblast fusion as a fundamental biological process, which can ultimately be leveraged to improve fusion efficiency in in vitro models and potentially be used as a novel therapeutic target for placental dysfunction in vivo.

## Methods

Unless otherwise stated, all cell culture materials and supplies were purchased from Fisher Scientific (Ottawa, ON) and chemicals from Sigma Aldrich (Oakville, ON).

### Cell culture—monolayers

BeWo human placental choriocarcinoma cells (ATCC; CCL-98) between passages 20–25 (for monolayers) or passages 4–6 (for spheroids) were cultured in 10% foetal bovine serum (FBS) and 1% antibiotic–antimycotic in Dulbecco’s Modified Eagle Media (DMEM). BeWo cells were seeded at ~ 120,000 cells/cm^2^ to form a continuous monolayer sheet of cells. Seeding density was adjusted for mechanical compression/tension experiments such that the final nuclear density after mechanical deformation of the substrate was comparable. Media was supplemented with 24 µM forskolin (CAS: 66575–29-9) to induce fusion, with media replaced every 24 h. For traction force microscopy (TFM) experiments, an excess volume of media was used to avoid disrupting the automated imaging for a media exchange.

### Cell culture—spheroids

A custom stamp block with a 1 mm cone at the surface was adapted from a previous technology^44^, and designed to fit a 48 well plate using Fusion 360 (Autodesk) and printed in PR57-K black prototyping resin (Colorado Photopolymer Solutions) using the Autodesk Ember DLP 3D printer. To create micropockets, Loctite AA3525 was used as an adhesive to attach polyacrylamide gels to the bottom of the well plate^51^. The adhesive was incubated for 1.5 h in RO water to limit toxicity, after which a polyacrylamide mixture corresponding to 25.6 kPa shear stiffness^52^ was added to the bottom of the well and covered immediately with the stamp block. After curing for 8 min, the block was removed and left in PBS solution containing 1% antibiotic–antimycotic for at least 3 days and with the solution changed every 24 h to remove any residual chemicals that might be harmful to cells. On the day of experimentation, microwells were exposed to 365 nm UV light for 1–2 h and then incubated in complete culture media. 50,000 cells were pipetted into the well containing the micropocket and the well plate was centrifuged at 400 RCF for 10 min, after which the well plate was incubated at 37 °C for 1–2 days to enable spheroids to form.

### Traction force microscopy

We have previously determined polyacrylamide hydrogel formulations that result in gels with stiffnesses that mimic native healthy placental tissue mechanical properties^33,53^. For TFM experiments, prepolymer solutions of the 3.9 kPa native placental stiffness containing 0.5% volume of 0.5 μm diameter carboxylate-modified fluorescent beads in PBS (FluoSpheres, Invitrogen, Catalog: F8812) were prepared as previously described^34^. To functionalize the gel surface, the gels were activated twice with 0.1 mg/ml of the photoactivable bifunctional crosslinker N-sulfosuccinimidyl-6-[4’-azido-2’-nitrophenylamino] hexanoate (sulfo-SANPAH, ProteoChem); and incubated with an excess amount of 80–100 µl of bovine Collagen I (0.1 mg/ml in phosphate buffered saline or PBS, Life Technologies) on a parafilm sheet at 4 °C overnight. Such TFM substrates prepared on 12 mm glass coverslips were attached to the bottom of a 24 well plate using a drop of cured polydimethylsiloxane (PDMS, 10:1 pre-polymer: curing agent, Dow Sylgard 184) and exposed to 365 nm UV light for at least 45 min to facilitate quick attachment and to prevent detachment of gels, after which gels were washed in sterile PBS and immediately used for experiments.

TFM substrates were incubated in complete DMEM for 2 h at 37 °C and then with BeWo cells, which were allowed to attach and form a uniform monolayer over 24 h. Cultures were induced to fuse by adding 24 µM of forskolin, and fluorescent/phase contrast images of the uppermost layer of embedded beads were captured every 30 min using an automated fluorescent microscope (20 × objective, EVOS m7000, ThermoFisher Scientific) over 48 h after induction.

### TFM image analysis

To analyse the collected TFM image datasets, template alignment, particle image velocimetry (PIV), and Fourier transform traction cytometry (FTTC) ImageJ plugins were used as per previous protocols^54^. Briefly, both stressed and relaxed fluorescent bead images were combined into a stack and aligned to account for experimental drift. The bead displacements were estimated by PIV (advanced) following an iterative procedure where the interrogation window was made progressively smaller (128 × 128 pixels, 64 × 64 pixels, 48 × 48 pixels; correlation threshold: 0.6) to produce an ultimate displacement field grid of ~ 14.8 μm × 14.8 μm. Traction force fields could then be reconstructed with the FTTC plugin using values: pixel = 0.3086 μm, Poisson’s ratio = 0.457, and Young's Modulus = 3900 Pa, along with default values for remaining inputs. This analysis was repeated for each 30-min interval image over 48 h using a custom ImageJ macro. The first time point at t = 0 (time of forskolin induction) was used as the reference image to determine the relative traction stresses that arise during forskolin-induced.

Fused syncytialized regions were identified by analysis of Syndecan-1^37,38^ immunofluorescent expression in fixed cultures after 48 h of forskolin induction. Only regions that were 75–80% fused were selected for analysis. These regions of interest (ROIs) were enlarged by 80 pixels, to empirically include 1–2 cells outside the region of fusion to include their influence on the stress patterns. A corresponding ROI of the same area but for a non-fused mononucleated region (25–30% fusion efficiency) was also selected for analysis. Since the precise timepoint(s) at which fusion occurs within each ROI is unknown, all time-points in the 48-h fusion window were analyzed for traction stresses, capturing the history of stresses at specific sites. This analysis therefore allows us to retroactively quantify stresses that had occurred during the fusion process. Using a custom Matlab code, traction stress vectors were then decomposed into radial stresses (oriented inwards/outwards to the areal centroid of the ROI), and tangential stresses (oriented perpendicular to the radial vector). For quantification purposes, the average radial and absolute tangential stresses from t = 12 to 48 h was calculated for each ROI to compare between syncytial vs. mononucleated regions.

### Uniaxial compression stimulation experiments

Uniaxial compression experiments were performed with a commercial mechanical stimulation platform (CellScale MechanoCulture FX) using a 24 well silicone membrane platform. The silicone platform was pre-stretched to 10% uniaxial strain, coated with 0.1 mg/ml bovine Collagen I by incubating at 37 °C for 2 h, seeded with cells in the stretched condition and left undisturbed for 24 h to form a sheet. Similar steps were performed on a separate static 24 well silicone membrane platform to be used as a control. A mechanical stimulation regimen consisting of 5% uniaxial compression over 6 h and no movement for 18 h was repeated twice for a total of 2 days, so that an overall 10% uniaxial compressive strain could be achieved. Media containing forskolin was changed daily. After 2 days of stimulation, cells were fixed, immunostained, and imaged.

### Equibiaxial compression and tension stimulation experiments

Both equibiaxial compression and tension experiments were performed using a custom iris-like stretchable device system that has been previously described in multiple stretching applications^39,55^. In order to establish a static control to compare against the commercial pre-fabricated stretchable dishes used for the stimulation experiments, a silicone membrane with similar properties to that of the stretchable dishes was replicated in a 35 mm petri dish by mixing equal parts of Silicone Elastomer Parts A and B (Elkem LSR-4305 Elastomer, Product Code: A-221–05; Factor II, Inc.) and cured in an oven at 70 °C for 2 h after degassing. The Young’s modulus of both the static control petri dishes and stretchable dishes were verified using rheometry (see Supplementary Methods S1). Both the stretchable dishes and the static control petri dishes containing silicone were washed with double distilled water (ddH_2_O) and treated with 30% sulfuric acid for 15 min at room temperature. Both platforms were again rinsed with ddH_2_O and then treated with 1% (3-aminopropyl) triethoxysilane (APTES) for 2 h in a 70 °C oven. Both platforms were again rinsed with ddH_2_O and stored in a 4 °C refrigerator for up to a month prior to use in experiments.

On the day of experimentation, both platforms were treated with 1% glutaraldehyde solution for 15 min at room temperature after which both platforms were rinsed with ddH_2_O, sprayed with 70% ethanol, and taken into a biological safety cabinet for sterility with subsequent steps. The stretchable dish was connected to a computer to be pre-stretched using a custom LabVIEW based software to 10% strain from the base minimum area (for compression experiments) or pre-stretched to the required base minimum area (for tension experiments). Both platforms were coated with 0.05 mg/ml Collagen I by incubating at 37 °C for 2 h. After rinsing with sterile PBS, cells were seeded in both platforms and left undisturbed up to 24 h to form a monolayer. Static control cultures were seeded at increased or decreased cell densities such that the number of cells per unit area after mechanical compression/tension was similar. A mechanical stimulation regimen consisting of 5% equibiaxial compression (or tension) over 6 h and no movement for 18 h per day was repeated twice for a total of 2 days, so that an overall 10% equibiaxial compressive (or tensile) strain could be achieved. Media containing forskolin was changed daily. At the end of 2 days, cells were fixed, the bottom of stretchable dishes were cut out with a scalpel, and then immunostained and imaged. Experiments were repeated for 3 independent stretchable dishes and static petri dishes with results normalized to the corresponding control for comparison. Due to limitations in the number of stretching platforms available, it was necessary that repeated experiments be performed at different passage numbers. Since passage number is known to affect BeWo cell fusion efficiency, a fold-based analysis was used to normalize fusion efficiency within each passage number.

### Immunostaining

At the end of uniaxial compression, equibiaxial compression/tension and TFM experiments, 8% (w/v) formalin in PBS was added to samples containing 50% remaining media to fix for 12–15 min at room temperature. Samples were then rinsed in PBS, permeabilized with 0.1% (v/v) Triton X-100 in PBS for 12–15 min, washed in PBS, blocked with 2.6% (v/v) goat serum, and incubated overnight at 4 °C with primary rabbit anti-Syndecan-1 antibody (HPA006185, 1:200 dilution) and primary mouse monoclonal anti-E-cadherin (ab1416, 1:200 dilution, Abcam) for fusion analysis. Secondary staining was performed with goat-anti mouse IgG H&L antibody (Alexa Fluor^®^ 594 red, 1:1000 dilution) and goat anti-rabbit IgG H&L antibody (Alexa Fluor^®^ 488 green, 1:1000 dilution, Invitrogen) for 3 h, and counterstained with Hoechst 33258 (5 µg/ml) for 2 h. For whole spheroids with all steps performed overnight at 4 °C, samples were fixed with 4% (w/v) formalin in PBS, permeabilized and blocked together in a buffer solution containing 0.5% Triton X-100 and 5% goat serum in PBS (v/v), primary stained with goat anti-Syndecan-1 antibody and mouse anti-human choriogonadotropin (β-hCG) antibody (#14–6508-82, 1:500 dilution in the buffer solution, ThermoFisher), and secondary stained as previously described. All samples were kept at 4 °C until image acquisition.

### Osmotic compression of 3D cultures

Spheroids were used for fusion-compression experiments under the combinatorial conditions of with/without forskolin and with/without dextran, where dextran (Mw = 500 kDa, Dextran Products; Polydex Pharmaceutical) was added separately as 25 mg or 50 mg of dextran per ml of complete DMEM media. Media was changed every 24 h and timelapse images at time points t = 0, 3, 6, 24, 30 and 48 h were acquired to assess compaction though compressive strains. After 48 h, samples were washed with PBS and then fixed overnight for immunostaining or histology to confirm findings (see Supplementary Methods S2).

### COMSOL modelling: determination of spheroid compression due to dextran

The isotropic compression applied on the spheroids due to the concentration of dextran used was calculated using a model similar to previous work^28^. In brief, disc-shaped polyacrylamide gels were fabricated by casting polyacrylamide prepolymer gel solution (18.75% v/v 40% Acrylamide, 2.70% v/v 2% Bisacrylamide, 68.40% v/v PBS, 0.15% v/v TEMED and 10.0% v/v APS) into 12 mm molds under a constant nitrogen gas flow for 15 min. After gelation, disc gels were washed with PBS 3 times and stored at 4 °C to allow the gels to swell. After 7 days, the rheological properties of disc gels were measured through rheometry (see Supplementary Methods S1). We have previously demonstrated that this dextran chain length formulation does not penetrate polyacrylamide gels^28^. The change in size of the gels due to dextran induced osmotic compression was measured using dextran solutions with concentrations of 25 mg/ml and 50 mg/ml in PBS. To do so, images of the disc bulk gel were acquired using the EVOS m7000 microscope at 4X magnification before and 30 min after addition of Dextran solution at 37 °C, and the corresponding change in diameter was calculated in ImageJ (NIH).

To quantify the compressive forces generated by the dextran, an inverse finite element simulation was made in COMSOL Multiphysics 6.0 (Comsol Inc., Burlington, MA, USA). Disc shaped geometries were generated in a 2D axisymmetric model, and a pressure boundary condition was set on the top and side while a roller condition was placed on the bottom surface. A free triangular mesh was generated with predefined extremely fine element size giving an average element quality above 0.9. Discs were prescribed linearly elastic properties with a Young’s modulus measured by rheometry (see Supplementary Methods S1) and a Poisson ratio estimated at 0.457 from literature^56^. Using the experimentally measured pre- and post-compression gel sizes, the applied pressure was determined.

### Microscopy

All samples of equibiaxial compression or tension experiments were imaged on an inverted fluorescent Olympus microscope (Olympus, IX73) outfitted with an sCMOS Flash 4.0 Camera and Metamorph software (version 7.8.13.0) for brightfield and corresponding fluorescence filters (blue for Hoechst 33,258, red for E-Cadherin and green for Syndecan-1). For TFM experiments, the uniaxial compression experiment, spheroid compression time lapse experiment and spheroid histology sections, samples were imaged on an inverted EVOS m7000 microscope for brightfield and corresponding fluorescence filters (blue for Hoechst 33258, green for Syndecan-1 and red for fluorescent beads or E-cadherin). Images were acquired at random locations at 20X magnification for stimulation experiments and spheroid sections, at fixed positions at 20X magnification stored in memory over 48 h at 30-min intervals for TFM experiments, and per well at 4X magnification over 48 h at time points t = 0, 3, 6, 24, 30 and 48 h for the spheroid compression time lapse experiment.

Whole spheroid samples were imaged on a customized SCAPE 2.0 light sheet microscopy system^57^ in a top-down format, outfitted with a water dipping objective lens (20X 1.0 NA) and an Andor Zyla 4.2 + CMOS camera. Samples were individually encased in 1% agarose (UltraPure™ Agarose, Invitrogen) in a 35 mm petri dish prior to imaging. Slices of the entire sample were obtained at 10 μm spacing under 9.3X magnification, using a 488 nm excitation laser with 100 ms exposure and 10 mW power at source settings (green for Syndecan-1) and a 561 nm excitation laser with 100 ms exposure and 8 mW power at source settings (red for β-hCG).

### Image analysis

All images were analysed in FIJI ImageJ (NIH). For fusion analysis, cells expressing Syndecan-1 or a colony containing 2 or more cells sharing the same membrane boundary expressing E-cadherin were considered to be fused. The total number of nuclei (T) was counted either manually or using the ParticleSizer plugin in ImageJ^58^ and used to calculate nuclear density over a fixed area of 0.34 mm^2^ whereas the number of nuclei in a fused syncytia (F), the number of syncytia (S) and the number of nuclei expressing Syndecan-1 (FS) were counted manually. Based on the usage of E-cadherin or Syndecan-1 expression for analysis, fusion efficiency was calculated as described below, based on similar techniques previously reported^34,37,59^:

1 $$Fusion \,Efficienc{y}_{\left(w/ E-Cadherin\right)} \left(\%\right)=\frac{F-S+1}{T} \times 100,$$

2 $$Fusion \,Efficienc{y}_{(w/ Syndecan-1)} \left(\%\right)=\frac{FS}{T} \times 100.$$

For analysis of fusion in spheroids, the laser illumination settings, camera capture settings, and brightness and contrast settings of all slices for all conditions were uniformly and consistently maintained for Syndecan-1 and β-hCG image collection respectively. To avoid sampling issues associated with selecting a single optical section of an intact spheroid, and to avoid out-of-plane light collection from affecting the analysis, we calculate the integrated fluorescent signal intensity for the entire spheroid, and normalized this against the volume of each spheroid (measured by analyzing the area of each spheroid in each slice of the imaging stack). This value was used as a proxy for fusion levels with arbitrary units, and was calculated as follows:

3 $$Fusion \left(AU\right)=\frac{Total \,Integrated\, Density}{Total\, Volume\, O f\, Spheroid}.$$

Compressive strains in timelapse images of spheroids subjected to forskolin and/or dextran compression was calculated by finding the effective change in diameter of the spheroid at each time point (assuming a spherical structure) compared to that at t = 0 h. Immunostained sectioned spheroid images were created by overlaying all channels (nuclei, Syndecan-1 and E-cadherin) with brightness and contrast values fixed independently for each channel.

### Statistical analysis

Statistical analysis was performed using JASP, version 0.16.3^60^. Independent Student’s *t*-test was used for the uniaxial fusion analysis and comparison of TFM stresses, one-way ANOVA with post-hoc Tukey’s test for pairwise comparisons was performed for equibiaxial compression and tension fusion and nuclear density analyses whereas two-way ANOVA with post-hoc Tukey’s test for pairwise comparisons was performed for spheroid fusion analyses. In all cases, p ≤ 0.05 was considered statistically significant.

### Schematics

Schematics for Figs. 2, 3, 4 and 5 were created with BioRender.com.

### Supplementary Information

Supplementary Information.

## Data Availability

The custom code (ImageJ Macro and MATLAB) utilized for TFM analysis as well as the raw images obtained for each of the experiment described in this work are accessible via the Open Science Foundation at: https://osf.io/23ypd/?view_only=5575a67edb264367af652025a6a4beba.

## References

[1] Griffiths SK, Campbell JP (2015). Placental structure, function and drug transfer. Contin. Educ. Anaesth. Crit. Care Pain.

[2] Tetro N, Moushaev S, Rubinchik-stern M, Eyal S (2018). The placental barrier: The gate and the fate in drug distribution. Pharm. Res..

[3] Blundell C (2016). A microphysiological model of the human placental barrier. Lab. Chip.

[4] Wang Y, Zhao S, Wang Y, Zhao S (2010). Chapter 3—Structure of the placenta. Vascular Biology of the Placenta.

[5] Langbein M (2008). Impaired cytotrophoblast cell-cell fusion is associated with reduced Syncytin and increased apoptosis in patients with placental dysfunction. Mol. Reprod. Dev..

[6] Parameshwar PK (2022). Engineered models for placental toxicology: Emerging approaches based on tissue decellularization. Reprod. Toxicol..

[7] Deval G, Boland S, Fournier T, Ferecatu I (2021). On placental toxicology studies and cerium dioxide nanoparticles. Int. J. Mol. Sci..

[8] Blundell C (2018). Placental drug transport-on-a-chip: A microengineered in vitro model of transporter-mediated drug efflux in the human placental barrier. Adv. Healthc. Mater..

[9] Zhu Y (2018). Placental barrier-on-a-chip: Modeling placental inflammatory responses to bacterial infection. ACS Biomater. Sci. Eng..

[10] Lee JS (2016). Placenta-on-a-chip: A novel platform to study the biology of the human placenta. J. Matern. Fetal Neonatal Med..

[11] Richardson L (2019). Fetal membrane organ-on-chip: An innovative approach to study cellular interactions. Reprod. Sci..

[12] Schuller P (2020). A lab-on-a-chip system with an embedded porous membrane-based impedance biosensor array for nanoparticle risk assessment on placental Bewo trophoblast cells. Sens. Actuators B Chem..

[13] Cao R, Wang Y, Liu J, Rong L, Qin J (2023). Self-assembled human placental model from trophoblast stem cells in a dynamic organ-on-a-chip system. Cell Prolif..

[14] Turco MY (2018). Trophoblast organoids as a model for maternal–fetal interactions during human placentation. Nature.

[15] Sheridan MA (2020). Establishment and differentiation of long-term trophoblast organoid cultures from the human placenta. Nat. Protoc..

[16] Haider S (2018). Self-renewing trophoblast organoids recapitulate the developmental program of the early human placenta. Stem Cell Rep..

[17] Castellucci M, Schepe M, Scheffen I, Celona A, Kaufmann P (1990). The development of the human placental villous tree. Anat. Embryol. (Berl.).

[18] Castellucci M, Kosanke G, Verdenelli F, Huppertz B, Kaufmann P (2000). Villous sprouting: Fundamental mechanisms of human placental development. Hum. Reprod. Update.

[19] Gauster M, Moser G, Orendi K, Huppertz B (2009). Factors involved in regulating trophoblast fusion: Potential role in the development of preeclampsia. Placenta.

[20] Huppertz B, Borges M, Chen EH (2008). Placenta trophoblast fusion. Cell Fusion: Overviews and Methods.

[21] Renaud SJ, Jeyarajah MJ (2022). How trophoblasts fuse: An in-depth look into placental syncytiotrophoblast formation. Cell. Mol. Life Sci..

[22] Jauniaux E, Poston L, Burton GJ (2006). Placental-related diseases of pregnancy: involvement of oxidative stress and implications in human evolution. Hum. Reprod. Update.

[23] Shyer AE (2013). Villification: How the gut gets its villi. Science.

[24] Varner VD, Gleghorn JP, Miller E, Radisky DC, Nelson CM (2015). Mechanically patterning the embryonic airway epithelium. Proc. Natl. Acad. Sci..

[25] Kim HY, Varner VD, Nelson CM (2013). Apical constriction initiates new bud formation during monopodial branching of the embryonic chicken lung. Development.

[26] Millay DP (2022). Regulation of the myoblast fusion reaction for muscle development, regeneration, and adaptations. Exp. Cell Res..

[27] Xu X-Y (2012). Differential effects of mechanical strain on osteoclastogenesis and osteoclast-related gene expression in RAW264.7 cells. Mol. Med. Rep..

[28] Lee W (2019). Dispersible hydrogel force sensors reveal patterns of solid mechanical stress in multicellular spheroid cultures. Nat. Commun..

[29] Lee W (2023). Ultrasoft edge-labelled hydrogel sensors reveal internal tissue stress patterns in invasive engineered tumors. Biomaterials.

[30] Boghdady C-M, Kalashnikov N, Mok S, McCaffrey L, Moraes C (2021). Revisiting tissue tensegrity: Biomaterial-based approaches to measure forces across length scales. APL Bioeng..

[31] Gjorevski N, Nelson CM (2010). Endogenous patterns of mechanical stress are required for branching morphogenesis. Integr. Biol..

[32] Rejniak KA, Kliman HJ, Fauci LJ (2004). A computational model of the mechanics of growth of the villous trophoblast bilayer. Bull. Math. Biol..

[33] Ma Z, Sagrillo-Fagundes L, Mok S, Vaillancourt C, Moraes C (2020). Mechanobiological regulation of placental trophoblast fusion and function through extracellular matrix rigidity. Sci. Rep..

[34] Ma Z (2019). Biomimetic micropatterned adhesive surfaces to mechanobiologically regulate placental trophoblast fusion. ACS Appl. Mater. Interfaces.

[35] Wong MK (2018). Extracellular matrix surface regulates self-assembly of three-dimensional placental trophoblast spheroids. PLoS One.

[36] Lyden TW, Ng AK, Rote NS (1993). Modulation of phosphatidylserine epitope expression by BeWo cells during forskolin treatment. Placenta.

[37] Prakash GJ, Suman P, Gupta SK (2011). Relevance of syndecan-1 in the trophoblastic BeWo cell syncytialization. Am. J. Reprod. Immunol..

[38] Slaby EM, Plaisier SB, Brady SR, Hiremath SC, Weaver JD (2024). Controlling placental spheroid growth and phenotype using engineered synthetic hydrogel matrices. Biomater. Sci..

[39] Majd H (2009). A novel method of dynamic culture surface expansion improves mesenchymal stem cell proliferation and phenotype. Stem Cells.

[40] Dietrich B, Kunihs V, Haider S, Pollheimer J, Knöfler M (2021). 3-Dimensional JEG-3 choriocarcinoma cell organoids as a model for trophoblast expansion and differentiation. Placenta.

[41] Montel F (2012). Isotropic stress reduces cell proliferation in tumor spheroids. New J. Phys..

[42] Li Y (2021). Volumetric compression induces intracellular crowding to control intestinal organoid growth via Wnt/β-catenin signaling. Cell Stem Cell.

[43] Monnier S (2016). Effect of an osmotic stress on multicellular aggregates. Methods.

[44] Zhao L, Mok S, Moraes C (2019). Micropocket hydrogel devices for all-in-one formation, assembly, and analysis of aggregate-based tissues. Biofabrication.

[45] Al-Nasiry S, Spitz B, Hanssens M, Luyten C, Pijnenborg R (2006). Differential effects of inducers of syncytialization and apoptosis on BeWo and JEG-3 choriocarcinoma cells. Hum. Reprod..

[46] Ort C, Lee W, Kalashnikov N, Moraes C (2021). Disentangling the fibrous microenvironment: Designer culture models for improved drug discovery. Expert Opin. Drug Discov..

[47] Li B, Lin M, Tang Y, Wang B, Wang JH-C (2008). A novel functional assessment of the differentiation of micropatterned muscle cells. J. Biomech..

[48] Zaccolo M (2009). cAMP signal transduction in the heart: Understanding spatial control for the development of novel therapeutic strategies. Br. J. Pharmacol..

[49] Okae H (2017). Derivation of human trophoblast stem cells. Cell Stem Cell.

[50] de Camps CC (2023). Compressive molding of engineered tissues via thermoresponsive hydrogel devices. Lab. Chip.

[51] Díaz-Bello B (2019). Method for the direct fabrication of polyacrylamide hydrogels with controlled stiffness in polystyrene multiwell plates for mechanobiology assays. ACS Biomater. Sci. Eng..

[52] Tse JR, Engler AJ (2010). Preparation of hydrogel substrates with tunable mechanical properties. Curr. Protoc. Cell Biol..

[53] Kılıç F (2015). Shear wave elastography of placenta: In vivo quantitation of placental elasticity in preeclampsia. Diagn. Interv. Radiol..

[54] Tseng Q (2012). Spatial organization of the extracellular matrix regulates cell-cell junction positioning. Proc. Natl. Acad. Sci. U. S. A..

[55] Rosenzweig DH (2012). Culture of primary bovine chondrocytes on a continuously expanding surface inhibits dedifferentiation. Tissue Eng. A.

[56] Takigawa T, Morino Y, Urayama K, Masuda T (1996). Poisson’s ratio of polyacrylamide (PAAm) gels. Polym. Gels Netw..

[57] Voleti V (2019). Real-time volumetric microscopy of in vivo dynamics and large-scale samples with SCAPE 2.0. Nat. Methods.

[58] Wagner T, Eglinger J (2017). Zenodo.

[59] Brown LM, Lacey HA, Baker PN, Crocker IP (2005). E-cadherin in the assessment of aberrant placental cytotrophoblast turnover in pregnancies complicated by pre-eclampsia. Histochem. Cell Biol..

[60] JASP Team. JASP (Version 0.16.3) [Computer Software] (2022).

